# Supplementary material for: Microwave-Assisted Synthesis of N/TiO2 Nanoparticles for Photocatalysis under Different Irradiation Spectra
Source: Nanomaterials (Basel). 2022 Apr 26;12(9):1473. doi: 10.3390/nano12091473 (PMC9104789; doi:10.3390/nano12091473)
Supplement: Supplementary file 1 [file nanomaterials-12-01473-s001.zip › nanomaterials-1682051-supplementary.pdf]

nanomaterials

Article

Microwave-Assisted Synthesis of N/TiO_2 Nanoparticles for Photocatalysis under Different Irradiation Spectra

Camilo Sanchez Tobon [1,*], Davor Ljubas [1,*], Vilko Mandić [2], Ivana Panžić [2], Gordana Matijašić [2] and Lidija Ćurković [1,*]

[1] Faculty of Mechanical Engineering and Naval Architecture, University of Zagreb, 10000 Zagreb, Croatia
[2] Faculty of Chemical Engineering and Technology, University of Zagreb, 10000 Zagreb, Croatia; vmandic@fkit.hr (V.M.); ipanzic@fkit.hr (I.P.); gmatijas@fkit.hr (G.M.)
* Correspondence: camilo.sanchez.tobon@fsb.hr (C.S.T.); davor.ljubas@fsb.hr (D.L.); lidija.curkovic@fsb.hr (L.Ć.)

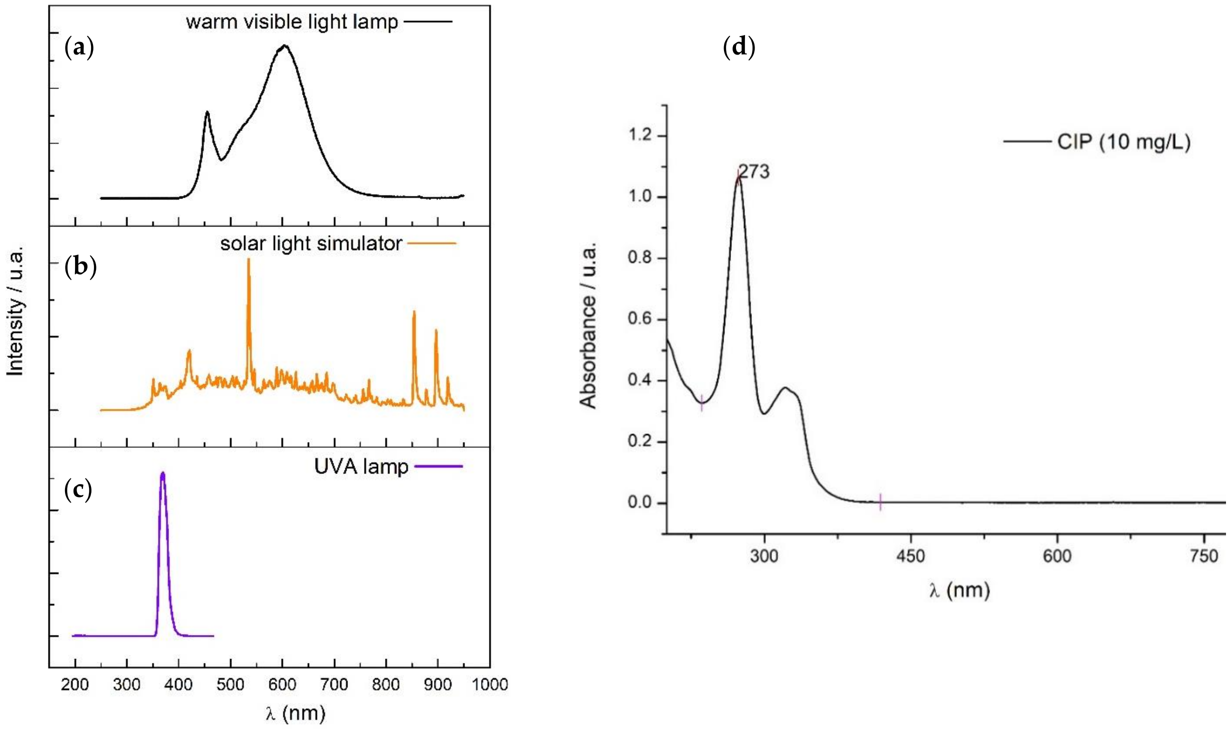

Figure S1. Radiation spectra of three different lamps: (**a**) warm visible light, (**b**) Solar light, and (**c**) UVA. (**d**) UV/Vis Absorbance spectrum of ciprofloxacin (CIP).

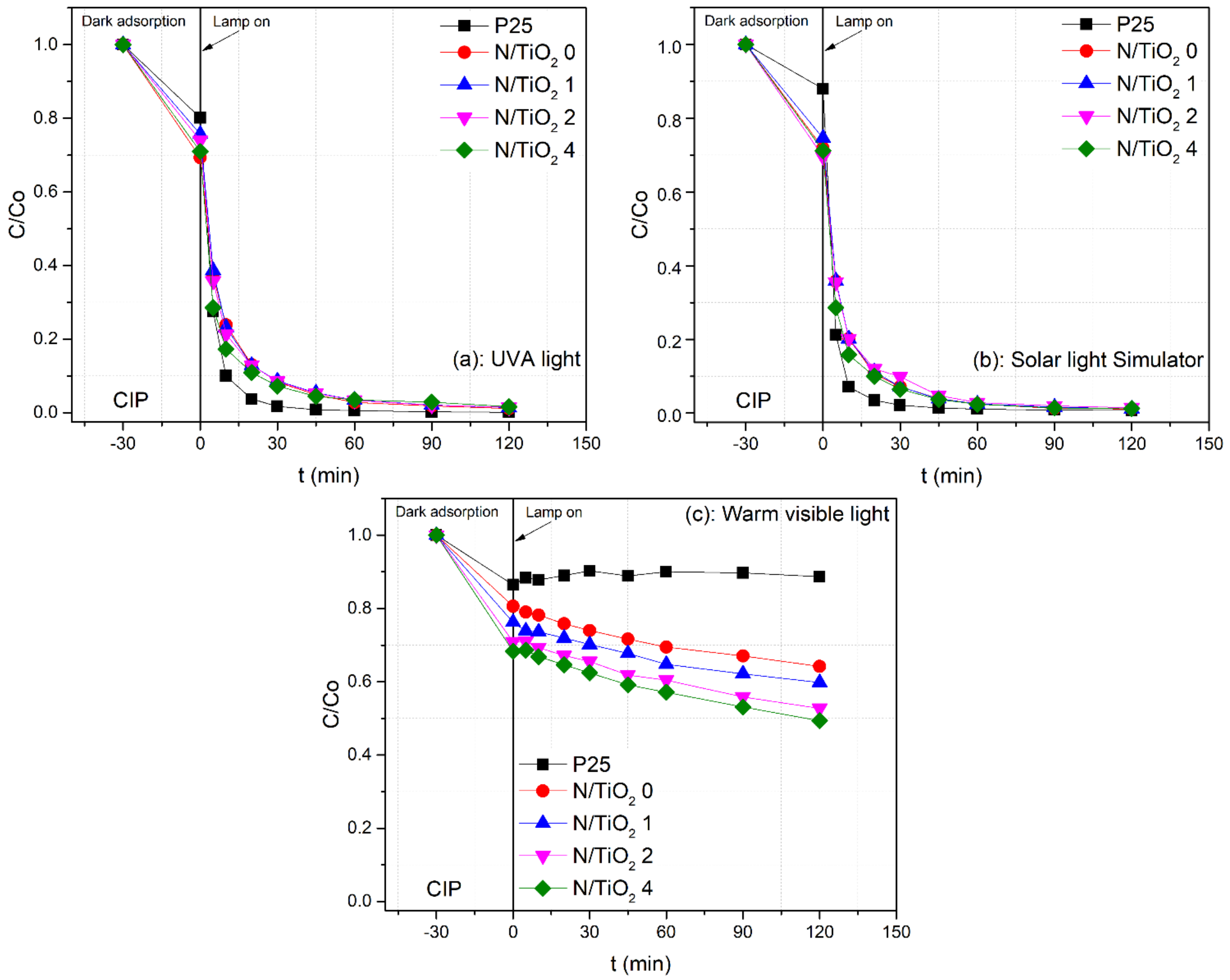

Figure S2. Photocatalytic degradation of ciprofloxacin by Degussa P25 TiO_2 and N/TiO_2 samples under (**a**) UVA light (lamp I), (**b**) Solar light simulator lamp (II), and (**c**) warm visible light (lamp III).

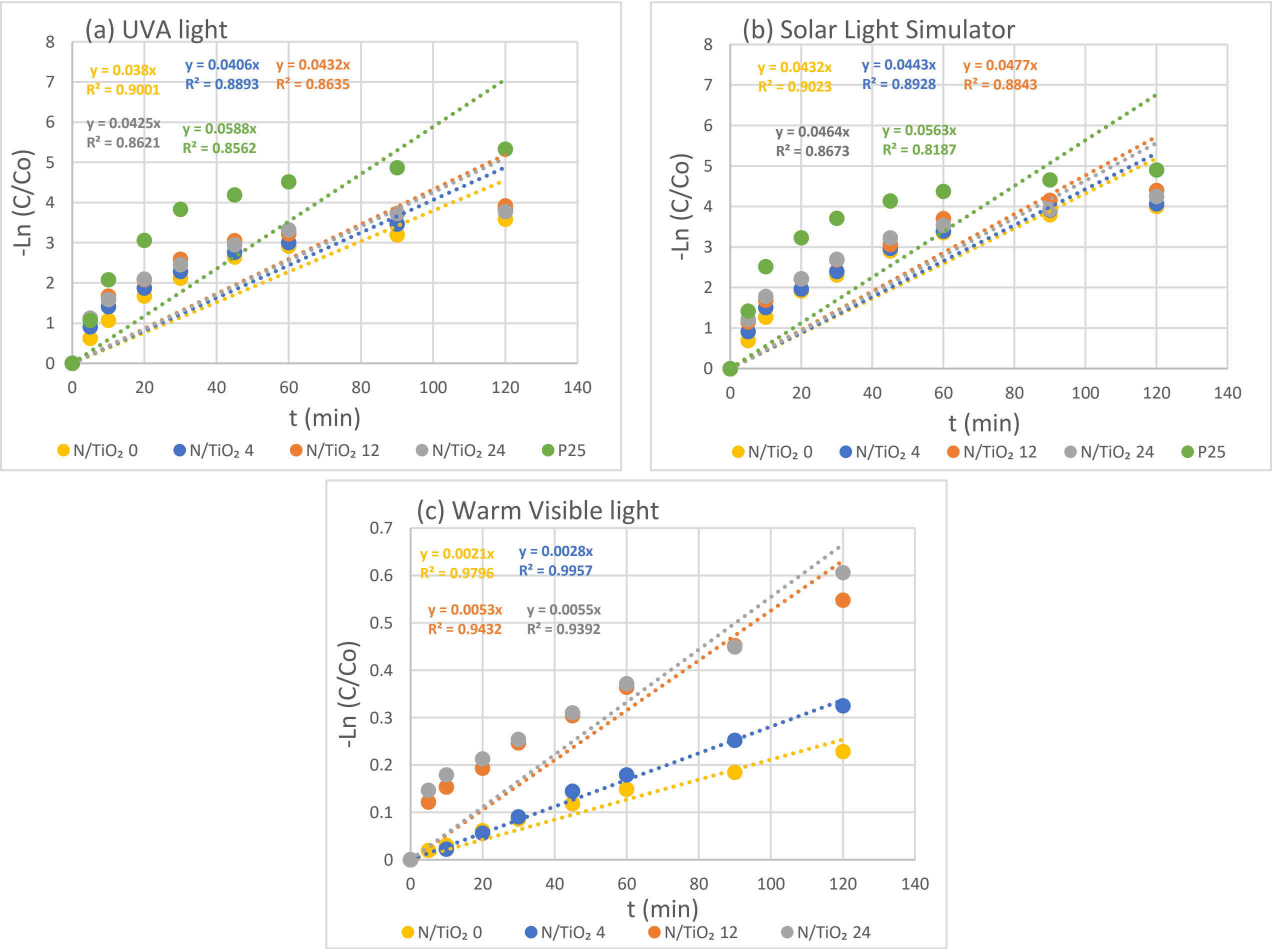

Figure S3. Pseudo-first-order kinetic rate of the photocatalytic degradation of ciprofloxacin by Degussa P25 TiO_2 and N/TiO_2 samples under (**a**) UVA light (lamp I), (**b**) Solar light simulator lamp (II), and (**c**) warm visible light (lamp III).

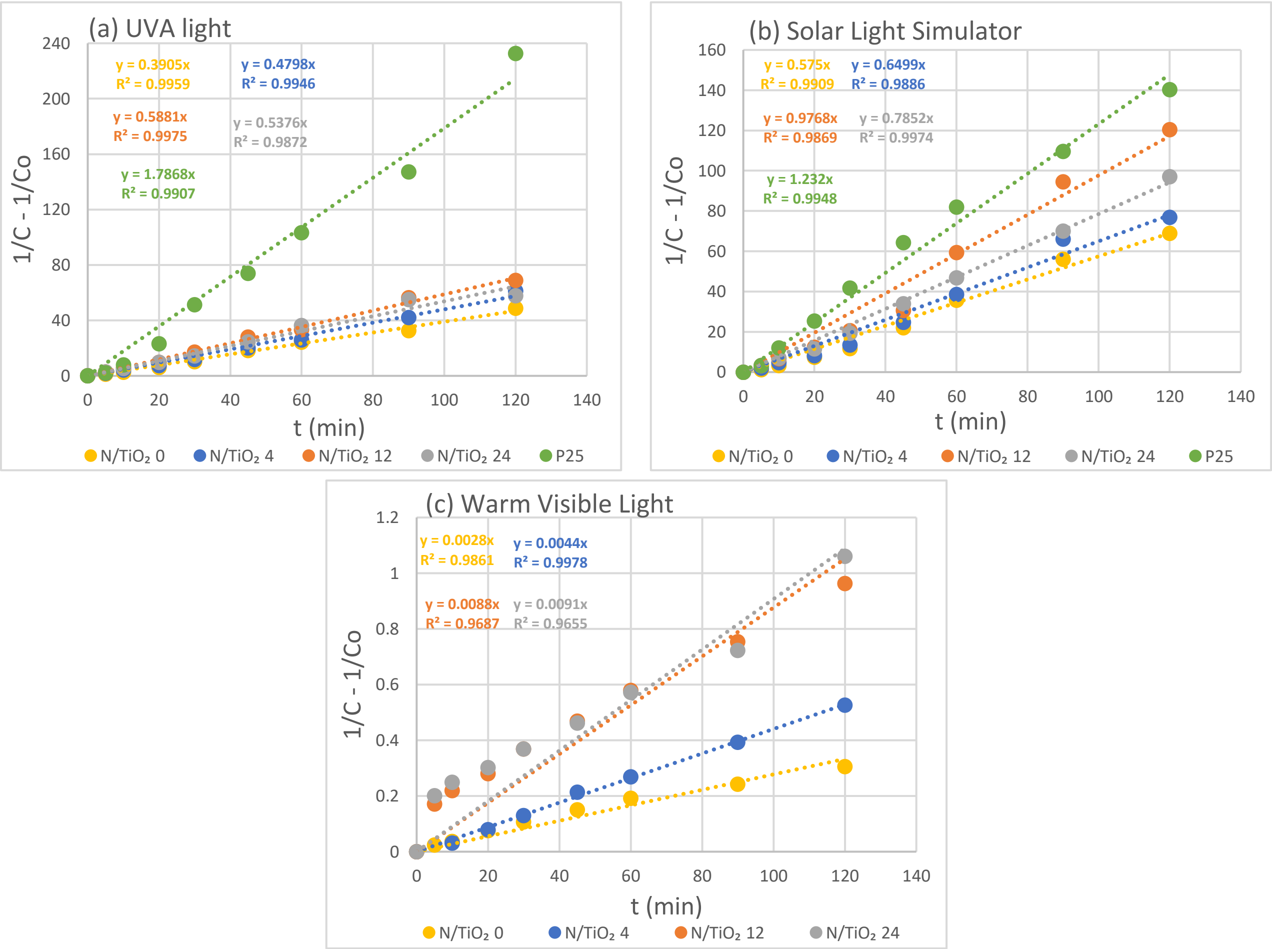

Figure S4. Pseudo-second-order kinetic rate of the photocatalytic degradation of ciprofloxacin by Degussa P25 TiO_2 and N/TiO_2 samples under (**a**) UVA light (lamp I), (**b**) Solar light simulator lamp (II), and (**c**) warm visible light (lamp III).
